# Automated Diagnosis of Optical Coherence Tomography Angiography (OCTA) Based on Machine Learning Techniques

**DOI:** 10.3390/s22062342

**Published:** 2022-03-18

**Authors:** Ibrahim Yasser, Fahmi Khalifa, Hisham Abdeltawab, Mohammed Ghazal, Harpal Singh Sandhu, Ayman El-Baz

**Affiliations:** 1 Faculty of Engineering, Mansoura University, Mansoura 35516, Egypt; ibrahim_yasser@mans.edu.eg; 2 Department of Bioengineering, University of Louisville, Louisville, KY 40292, USA; fakhal01@louisville.edu (F.K.); hisham.abdeltawab@louisville.edu (H.A.); harpal.sandhu@gmail.com (H.S.S.); 3 Electrical and Computer Engineering Department, Abu Dhabi University, Abu Dhabi P.O. Box 59911, United Arab Emirates; mohammed.ghazal@adu.ac.ae

**Keywords:** diabetic retinopathy (DR), optical coherence tomography angiography (OCTA), convolutional neural networks (CNN), image encryption, security analysis

## Abstract

Diabetic retinopathy (DR) refers to the ophthalmological complications of diabetes mellitus. It is primarily a disease of the retinal vasculature that can lead to vision loss. Optical coherence tomography angiography (OCTA) demonstrates the ability to detect the changes in the retinal vascular system, which can help in the early detection of DR. In this paper, we describe a novel framework that can detect DR from OCTA based on capturing the appearance and morphological markers of the retinal vascular system. This new framework consists of the following main steps: (1) extracting retinal vascular system from OCTA images based on using joint Markov-Gibbs Random Field (MGRF) model to model the appearance of OCTA images and (2) estimating the distance map inside the extracted vascular system to be used as imaging markers that describe the morphology of the retinal vascular (RV) system. The OCTA images, extracted vascular system, and the RV-estimated distance map is then composed into a three-dimensional matrix to be used as an input to a convolutional neural network (CNN). The main motivation for using this data representation is that it combines the low-level data as well as high-level processed data to allow the CNN to capture significant features to increase its ability to distinguish DR from the normal retina. This has been applied on multi-scale levels to include the original full dimension images as well as sub-images extracted from the original OCTA images. The proposed approach was tested on in-vivo data using about 91 patients, which were qualitatively graded by retinal experts. In addition, it was quantitatively validated using datasets based on three metrics: sensitivity, specificity, and overall accuracy. Results showed the capability of the proposed approach, outperforming the current deep learning as well as features-based detecting DR approaches.

## 1. Introduction

Diabetic retinopathy (DR) is among several retinal diseases that represent major public health threats, which can lead to vision loss [1,2]. Diabetes mellitus is a metabolic disease characterized by hyperglycemia, and diabetic retinopathy is one of the cardinal late-stage organic manifestations of the disease. Persistent hyperglycemia causes microvascular damage in the retina through a number of mechanisms, leading to pericyte loss, endothelial damage, and ultimately capillary permeability and/or dropout. As a result, the eye develops vascularanomalies, such as neovascularization on the surface of retina in the advanced form of the disease, called proliferative diabetic retinopathy (PDR); however, these new vessels are incompetent and tend to haemorrhage or scar [3]. Although there are no vision alterations in the early stages of DR, it eventually leads to vision loss [4,5]. As a result, early detection and treatment of DR can delay or prevent diabetic-related blindness [6].

In the International Clinical Diabetic Retinopathy Disease Severity Scale, DR is classified as either proliferative (PDR) or non-proliferative (NPDR). The non-proliferative DR (NPDR) kind is divided into categories: (a) mild NPDR, in which there is no alteration in vision and the retina has fewer microaneurysms; (b) moderate NPDR, which has more microaneurysms than mild NPDR but is less severe than Severe NPDR; and (c) severe NPDR, in which patients have obvious intraretinal microvascular abnormalities (IRMA), confirmed venous bleeding in two or more quadrants, and multiple intraretinal haemorrhages in all four quadrants. Many blood vessels are blocked in severe NDPR, which induces abnormal growth factor production. In proliferative DR (PDR), patients with vitreous/preretinal and neovascularization disease are at high risk of irreversible blindness without sufficient treatment, hence its designation as advanced disease [7].

The algorithms for diagnosis are dependent on the retinal medical imaging techniques, that can be categorized as non-invasive or invasive image techniques. Indocyanine green angiography (ICGA) and fluorescein angiography (FA) are invasive methods that require 10–30 min of imaging and intravenous dye administration. They show dynamic imaging of blood flow through retinal vessels in 2D images [8,9]. Non-invasive approaches, on the other hand, include OCT angiography (OCTA) and optical coherence tomography (OCT) [10,11]. OCTA is a technique that is used to acquire angiographic information non-invasively without the need to use dye [12]. In most cases, to correctly portray vessels through different segmented parts of the eye, OCTA uses the backscatter of laser light from the surface of moving red blood cells, and that may be more accurate in detecting microvascular changes in retinal disorders than standard FA [13].

Several studies in the literature have investigated using FA to diagnose diseases in the posterior segment of the eye [12]. Despite that, FA has some limitations, such as its inability to visualize different levels of major capillary networks separately. This is because FA is unable to differentiate deep capillary plexus (DCP) from superficial capillary plexus (SCP). Additionally, it is hard to use FA in obtaining enhanced images of perifoveal capillaries because it has a challenge in focusing images when macular edema is present [10]. Moreover, FA is an invasive, time-consuming and relatively expensive modality, which makes it not ideal for regular use in clinical settings. Fluorescein dye is known to be safe; however, its side effects include nausea, allergic reactions and anaphylaxis in some rare cases [14].

Artificial intelligence (AI) consists of a set of branches, including the machine learning (ML) branch, where based on frequent exposure to labelled datasets, algorithms learn to classify data, such as medical images. Medical imaging offers a plethora of applications for ML, and the area has recently flourished in ophthalmology, with a retinal imaging focus. Image analysis and diagnosis, on the other hand, are not the most important aspects of machine learning in medicine. These methods can be used to analyze a variety of data types, including clinical data and demographic. This paper’s goal was to utilize ML techniques and OCTA image data to build a computer-aided diagnostic (CAD) that automates DR diagnosis. The following are the most important contributions of this work:

- We propose a novel CNN model for OCTA scans, for tissue classification by exploiting multiple contrasts of OCTA.
- The method is based on a combination of three channels between gray, binary, and distance map to enhance the DL system ability to to capture both appearance and morphological markers of the retinal vascular system.
- Our system employs multi-scale levels to include the original full dimension images as well as sub-images extracted from the original OCTA images. This allow the CNN to capture significant features and increases its ability to distinguish DR from the normal retina.
- Evaluation has been conducted using in-vivo data and has been compared with DL-based methods as well as hand-crafted based approaches.

This paper is structured as follows: In Section 2, a general overview of existing OCTA classification is discussed followed by details of the proposed OCTA classification system in Section 3. In Section 4, experimental results for the proposed OCTA model are presented including classification performance metrics. Finally, the concluding remarks and suggested future works are given is Section 5.

## 2. Related Work

Computerized image analysis of retinal images is a hot topic in the scientific community. Many strategies for automatically classifying the severity of DR have been presented. Deep learning (DL) is a class of ML techniques which allows computational models made up of different processing layers to learn to represent data. DL has proven its superiority in providing a huge workforce and financial resources, and achieving high accuracy in many areas compared to traditional methods [15,16,17,18]. Through its strong diagnostic performance in detecting various pathological conditions, deep learning has been applied, mainly fundus images and to the examination of major eye diseases such as DR, age-related macular degeneration and glaucoma, which either depend on well-established guidelines or require long-term follow-up [19,20,21,22,23,24,25]. The convolutional neural network (CNN) has become the most extensively used technique for retinal images in particular and image classification problems in general. Most prior research used color fundus images to segment retinal blood vessels because there were few studies on OCTA image processing, OCTA being a relatively new modality. Heisler et al. [26] for example, used single data types to build the component of neural networks, which were fine-tuned using pretrained DenseNet, ResNet50, and VGG19 architectures. They studied the role of ensemble deep learning in classifying DR from OCTA images and co-registered OCT images of the retinal layers. For the stacking and majority soft voting approaches, ensemble networks built with the four fine-tuned VGG19 architectures obtained accuracies about 0.90 and 0.92, respectively. This research supports CNN’s ability to accurately diagnose DR in OCTA, but it does have several drawbacks, as using ensemble learning methods, for example, considerably increases the computational cost because it necessitates the training of several networks.

Eladawi et al. [27] developed a CAD system for DR diagnosis utilizing OCTA that includes retinal vessel (RV) segmentation, image–derived markers, and an SVM-based classification. Based on a stochastic approach, the system describes the appearance of blood vessels at different levels (“deep” and “superficial”) for diabetic and normal cases using a joint Markov-Gibbs random field (MGRF) model. Based on the biomarkers extracted from OCTA scans, their approach can diagnose various pathologies of choroid and retina. The image without the GGMRF and RDHE models has an AUC of 56.71%, a VVD of 58.33%, and a DSC of 54.56. While AUC was 96.18 percent, VVD was 7.95% and DSC was 96.04% for the image with enhancing stage. Le et al. [28] employed a deep-learning CNN architecture and VGG16 for automated OCTA classification using transfer learning. For training and cross-validation, a dataset of 131 images (75 DR, 24 diabetic without DR [non-DR], and 32 healthy) was employed. With the last nine layers retrained, the CNN architecture produced the greatest results. The retrained classifier’s cross-validation specificity, sensitivity, and accuracy for distinguishing between DR, non-DR, and healthy eyes were 90.82%, 83.7%, and 87.27%, respectively. The CNN can provide a prediction, but a physician will have no idea how the CNN arrived at that prediction. Thus, the lack of interpretability is one of this study’s flaws.

Nagasato et al. [29] utilized SVM and DL with a radial basis function kernel to create a nonperfusion area (NPA) automatic detection of retinal vein occlusion (RVO) in OCTA images. They examined the diagnostic ability of the seven ophthalmologists, SVM, and DNN (average required time, specificity and sensitivity). For discriminating NPA from normal OCTA images with RVO-OCTA images using the DNN, the mean specificity, sensitivity and AUC were 97.3%, 93.7%, and 98.6%, respectively. Average time to produce a diagnosis was 176.9 s. However, the study had some drawbacks, the 3×3 mm scan area was insufficient to detect the full NPA associated with RVO. Furthermore, they solely compared OCTA images between RVO and normal eyes, excluding the other diseases of the retina. Alam et al. [30] developed a DL-based framework for automated artery-vein (AV) classification in OCTA. On the test data, the AV-Net had an average accuracy of about 86.75 percent (86.71% for artery and 86.80% for vein), an F1-score of 82.81% and a mean IOU of 70.72 %. Because there are substantial areas of misclassification, such as at vessel cross points, this study suffers from some limitations. Díaz et al. [31] utilized a variety of morphological operators to select the FAZ candidates on the images of OCTA projection using FOVs two types. The method uses a combination of image processing algorithms to first identify the region in which the FAZ is located, then extract its precise contour. The proposed approach obtained an accuracy (Jaccard index) for diabetic OCTA images about 0.93 (0.83) and for healthy participants about 0.93 (0.82). Patients lacking various important illnesses that impact retinal vascularity are one of the drawbacks of design the research that include image datasets.

Kim et al. [32] used wide-field swept source OCTA (SS-OCTA) to construct the usefulness of semiautomated diagnostic for microvascular parameters for rating the DR severity with a perspectives variety. This study categorized 235 diabetic eyes into five categories: proliferative DR (PDR), severe NPDR, moderate NPDR, mild non-proliferative retinopathy (NPDR), and diabetes with no retinopathy (no-DR). The capillary NPA, vessel density (VD), and FAZ metrics were all measured. For grading severe NPDR from PDR, moderate from severe NPDR, mild from moderate NPDR, and no-DR, the NPA cutoff values were 21.4% (AUC: 0.90), 9.3% (AUC: 0.94), 4.7% (AUC: 0.94), and 3.7 percent (AUC: 0.91), respectively. The fundamental disadvantage of this study is that projection artefacts induced by bleeding or vitreous opacity might obscure normal microvasculature, resulting shadowing of the NPA. Ong et al. [33] developed a strategy based on the length of DCP skeletonized vessels. The deep capillary plexus (DCP), middle (MCP), and superficial (SCP) segments of OCTA slabs were segmented and thresholded using a new approach depending on DCP skeletonized vessel length. After adjusting for imaging quality and age, the adjusted flow index (AFI), parafoveal VD, and FAZ area from the vascular length density (VLD) of the SCP, as well as all three capillary layers, were compared between each DR severity category. The results showed that the values of AUC were moderate (0.731-–0.752), with specificity ranging from 57.1% to 64.3% and sensitivity ranging from 83.3% to 88.9%. One of the study’s drawbacks is the large racial differences between study groups as well as its insufficient powering for the DCP, which may have reduced the ability to resolve real variations in DCP parameters across groups. Alibhai et al. [34] proposed a semiautomatic, custom software method for eye scans with various degrees of DR or without DR for quantifying regions of nonperfusion capillary for OCTA classification. In eyes with proliferative DR, the mean percentage of nonperfused region was 8.5% (with 95% confidence interval CI: 5.0–14.3), in nonproliferative DR eyes, 2.1 percent (95 percent CI: 1.2–3.7), and in eyes without DR was 0.1 percent (with 95 percent CI: 0.0–0.4). The limitation of this study was that the sample size was modest, with only a few individuals having NPDR severe or moderate, necessitating that all eyes with NPDR be combined for statistical analysis purposes.

In summary, various algorithms and techniques have been developed and introduced for OCTA classification. Most of the previous techniques in the literature, however, have some limitations, such as: (1) Current discriminative methods suffer from insufficient features that can represent the OCTA problem, leading to lower accuracies. (2) Current generative methods suffer from the computationally expensive registration tasks. In addition, the built atlas may not represent well the image population. (3) Current deep learning methods suffer from the computationally expensive cost for training the CNN layers. In addition, the selection of the best number of layers and the best number of neurons per each layer is still an open research problem.

According to current surveys of DR works and the above literature survey, practically all examinations of retinal blood vessels are conducted using fundus imaging, a technique that lacks depth information. Furthermore, generality present DR-CAD systems begin with a threshold-based segmentation method, that may limit the diagnostic system’s specificity and sensitivity due to the error of segmentation. Furthermore, most of the published studies concentrated on examining retinal layers in OCTA images without taking into consideration in the retinal vascular system changes. Finally, several present DR-CAD systems make decisions based on widely extracted features, that may not be sensitive enough to detect DR early on.

To address the aforementioned problems, we present a comprehensive DR-CAD system that relies its diagnosis on newly derived features from the retinal blood vessels’ spatial structure and appearance. This improvement allows us to collect more imaging features of the retina, which improves classification accuracy and reduces noise. On the same cohort dataset, the overall framework was evaluated and compared to comparable studies.

## 3. The Proposed Classification Systems

**Figure 1:**
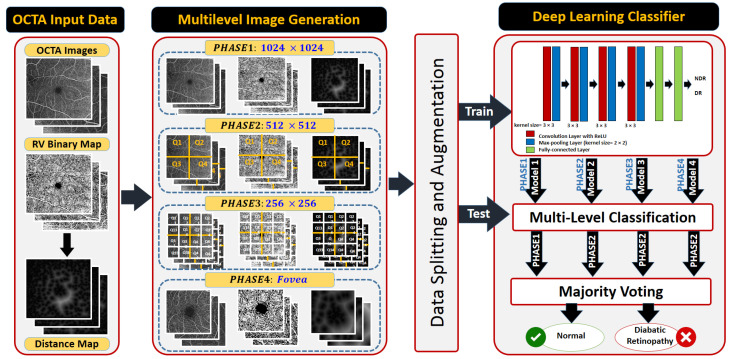
A schematic diagram of deep learning-based optical coherence tomography angiography pipeline.

A non-invasive, automated, early DR classification system from OCTA images is developed, and Figure 1 summarizes the proposed system’s essential steps. The proposed pipeline is composed of four parallel analysis phases. Essentially, we propose a multi-scale framework at which the OCTA images are analyzed at different scales. DR affects the width of vessels, thus it is in our best interest to present this for the network, because it is a direct feature correlated to DR disease. The input to the system contains three sources of information from which a multi-layer input image is constructed as an input to the classification system: the original superficial OCTA images, the original greyscale image, a binary map of its segmented retinal vasculature (RV) network and a distance map of blood vessels. The purpose of the segmented RV and its distance map is to introduce some sort of network’s attention as indicated by the reviewer to help improving the performance. Furthermore, we have used multi-scaled input (different input size) to help the network extract more local features from the greyscale channel. The retinal blood vessel structure is segmented using our previously developed segmentation approach [27].

From the combined images, multiscale inputs are constructed as inputs for different phases in the pipeline. Namely; the first phase provides a more global retinal features using full-sized images (i.e., 1024 × 1024) that is fed to a deep fully CNN. Smaller-sized images are used in the other phases for more local features extractions. Particularly, the full-sized OCTA image is split into equally-sized four quarters (i.e., 512 × 512 each) in the second phase, and into sixteen equally-sized parts (i.e., 256 × 256 each) in the third one. The last phase of our system is dedicated to extracting deep features around the fovea as a 512 × 512 window centered around the fovea is extracted and used to train and test another CNN. Individual CNN outputs are combined, and a soft voting method is utilized to combine the prediction scores of the individual networks for obtaining the final diagnosis.

### 3.1. RV Segmentation

**Figure 2:**
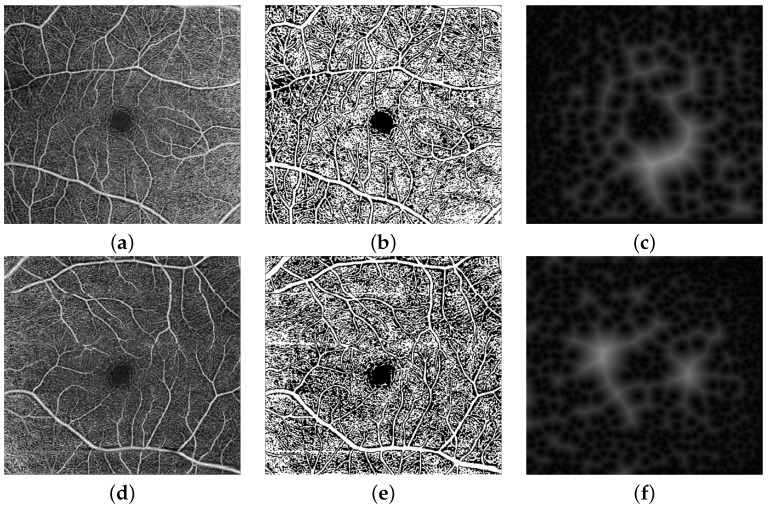
OCTA image example input data for DR and NDR: (**a**,**d**) the original superficial OCTA, (**b**,**e**) a binary map of the retinal vessels (RV), and (**c**,**f**) distance map of OCTA images.

The input to the CNN is a 3-channel image in which the second channel contains the binary map of the RV. As a result, for diabetic and normal instances, our pipeline first segments blood vessels in the deep and superficial compartments. Preprocessing, i.e. contrast enhancement and noise reduction, is first applied to the OCTA scans. This is achieved by using the RDHE algorithm [35] to ensure that the image’s grey levels are regularly distributed by altering each pixel’s intensity (grayscale) value based on the values of nearby pixels. After that, the generalized Gauss-Markov random field (GGMRF) model is used to reduce noise while preserving image detail [27]. Second, the vasculature was segmented from background using a combined Markov-Gibbs random field (MGRF) model. This combines a probabilistic “prior appearance” model of OCTA with spatial interaction (smoothness) and intensity distribution of different image segments. To overcome the poor contrast blood vessels and certain other retinal or choroidal tissue, the 1st-order intensity model, in addition to the higher order MGRF is employed to consider spatial information. Lastly, we enhanced the segmentation by applying a 2D connectivity filter to extract connected regions. Figure 2b,e shows two example RV Binary Map images with inadvertent contrast changes in various image regions.

In addition to the grayscale values and the RV binary maps, we also incorporate a distance-map-based image descriptor as the third channel of the input image to be analyzed. Namely, a signed distance map for the points of an object-background, or binary image, and is represented by the zero-level set, $B_{t}=\left\{p:\;p\in R;\;\Phi \left(p,t\right)=0\right\}$, of higher-dimensional function, Φ(p,t), on the lattice R, as follows:

$$\Phi \left(p,t\right)=\left\{\begin{array}{ccc} d(p,B_{t}) & \mathrm{if} & p\;\;\mathrm{in}\mathrm{the}\mathrm{interior}\mathrm{of}B_{t},\\ 0 & \mathrm{if} & p\in B_{t},\mathrm{and}\\ -d(p,B_{t}) & \mathrm{if} & p\;\;\mathrm{exterior}\mathrm{to}B_{t} \end{array}\right.$$

where $d\left(p,B_{t}\right)=\min_{b\in B_{t}}∥p-b∥$ is the distance from the point p to the surface $B_{t}$, as shown in Figure 2c,f.

### 3.2. Multilevel Image Generation

**Figure 3:**
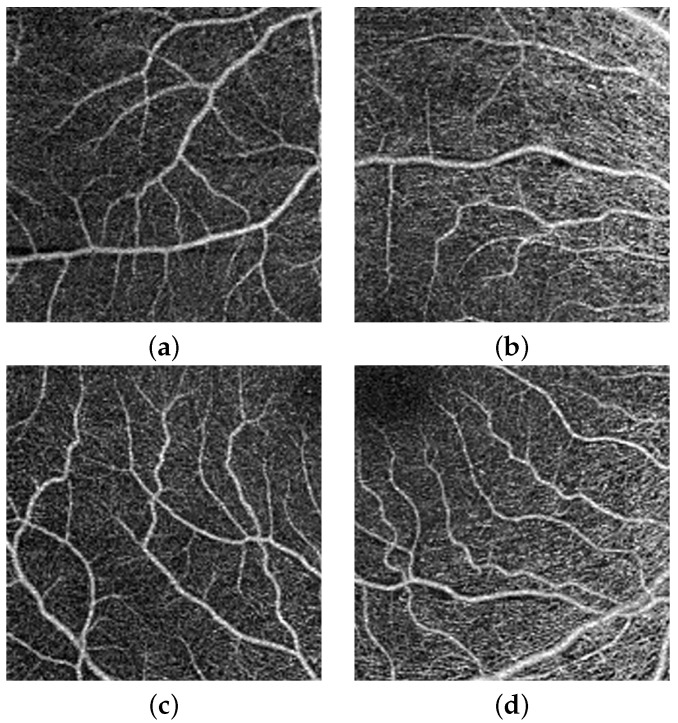
The four parts of OCTA image with equal size (512 × 512): (**a**) upper left, (**b**) upper right, (**c**) lower left, and (**d**) lower right quarter.

**Figure 4:**
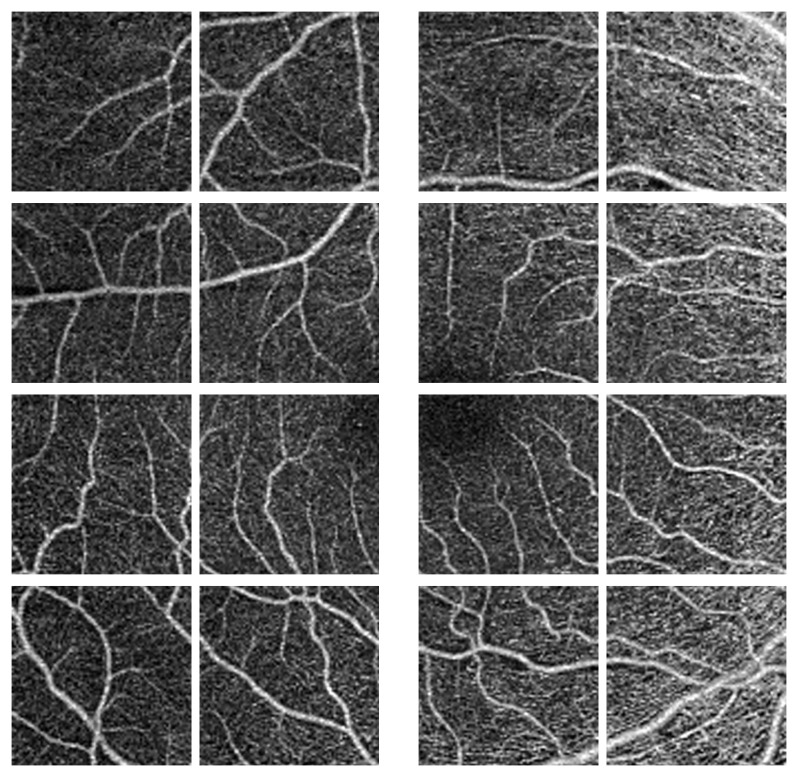
Normal OCTA image splitting for 16 equal size (256 × 256) sub-images.

**Figure 5:**
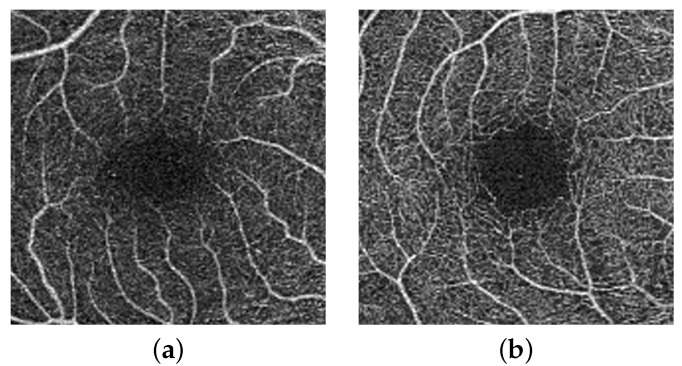
OCTA fovea zone with size (512 × 512); (**a**) DR, and (**b**) NDR.

The second stage of our proposed pipeline is generation of multi-scale images i.e., 512 × 512, and 256 × 256, shown in Figure 3 and Figure 4. The main idea behind this is that with smaller size will (1) avoid the inclusion of redundant surrounding vessel pixels and (2) emphasis local features and thus enhance the CNN learning process. According to previous research, the foveal region and FAZ are affected by various retinal diseases [36]. Thus, the area around the fovea includes features that can discriminate between normal and diseased subjects. To benefit from this and provide more accurate diagnosis, a more focused image around the center of the original image that includes the fovea is extracted, cropped (zone with size 512 × 512), and used as another level for diagnosis. Figure 5 shows cropping of the fovea in a diabetic patient versus a healthy control.

### 3.3. Deep Learning Classifier

**Figure 6:**
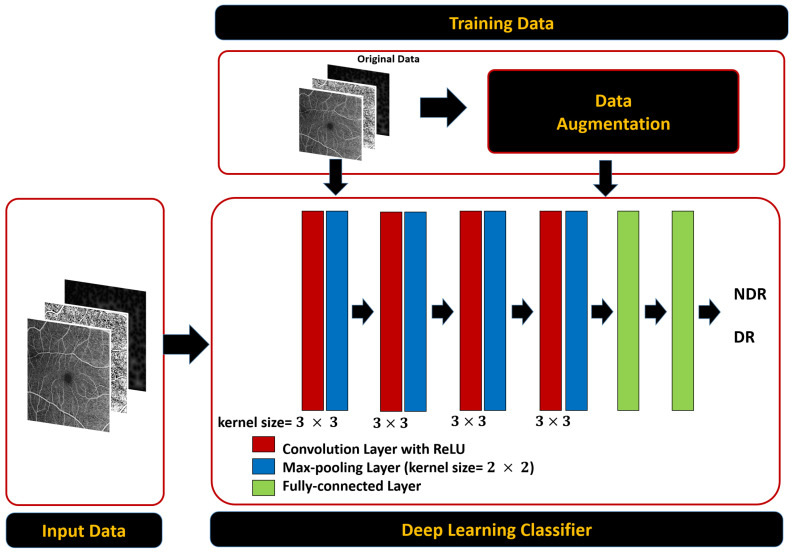
Schematic diagram of the proposed CNN with multi-input with size 1024 × 1024 that shows the design and the layers.

Our CNN architectures in Figure 6 were built using a succession of convolutional blocks, each of which had two convolutional layers followed by a max-pooling layer. Subsequent to these was a pair of fully connected layers and finally a soft-max output layer. The convolutional layers extract low-level feature maps comprising the trainable kernels’ responses to the input objects. Because we employed a filter group, each convolutional layer created a large number of feature maps. Filters with a size of 33, a stride of 1, and rectified linear unit (ReLU) activation functions were used in our design. In max-pooling layers, the feature maps spatial dimensions were lowered by a factor of two. The most significant features were maintained in the max-pooling layers, while the less important ones were removed. Additionally, max-pooling layers lowered computational cost and training time. In max-pooling layers, we used a stride of 2. In total, each CNN had four max-pooling layers and eight convolutional layers. For the four-way classification, the two fully connected layers had twelve and four neurons, respectively. The soft-max output layer translates the fully connected layer’s activation into class membership probabilities. The input patch is labeled with the class corresponding to the output neuron with the greatest probability. Our CNN model, with a total of 63,095,922 trainable parameters, is summarized in Table 1. Training used a 0.3 dropout rate and 0.001 learning rate. To find the optimal set of hyper-parameters, such as the number of layers, the nodes number in each layer (range: 10–100), L2 regularisation (range: 103–106), sparseness control parameters (range: 1–20), and the sparsity parameter (range: 0.05–0.9), grid search algorithm was used with the reconstruction error as the metric to optimise. The same principles can be applied to different patch sizes as well. Each CNN was trained by minimizing cross-entropy loss between ground truth labels and predicted class probabilities. The following is the definition of cross-entropy loss:

(1) $$LBCE=-\sum_{i=0}^{2N_{b}-1}y_{o,i}\log P_{o,i}$$

where *N* is the number of classes, $y_{o,i}$ is a binary indicator (0 or 1) which indicates the correct classification that observation o belongs to class i. The probability that observation o belongs to class i is given by $P_{o,i}$. In the convolutional and fully connected layers, a drop out with a rate of 0.2 was utilized to avoid network overfitting.

The usage of these architectures has two significant advantages, as we have shown in this paper. First, fine-tuning the network’s weights using the newly labeled images and pre-trained deep CNNs could result in enhanced performance metrics and a potential reduction in training resources such as memory, compute operations, and time. Second, even in a training from scratch scheme, the improved architecture and design of these CNNs may ensure greater performance ratios. For grading, we used a multi-level classification for classifying the inflammation of vitreous region into two classes (DR, NDR).

With the image’s distribution, the entire data set was separated randomly into two groups: training set and testing set, in addition to using the validation set to keep track of training process epochs. Table 1 shows the number of epochs that outperformed the in terms of accuracy in DR and NDR classes with the validation set. After the selection of the hyper-parameters, the proposed system was trained and tested using 91 cases of 55 DR and 36 NDR using a five-fold cross validation. The data sets are divided into 80% for training and 20% for testing. Throughout all the multi-level experiments, the test sets were identical.

## 4. Experimental Results

The OCTA scans were collected using a ZEISS AngioPlex OCT angiography machine. The AngioPlex OCT produces five different blood vessel maps. The deep and superficial retinal maps with pixels size of 1024 × 1024 were used to test our proposed CAD system. The images were captured on sections of 512 × 512 and are centered on the fovea. Every image was classified as to DR severity by a board-certified ophthalmologist. We considered two categories, DR and normal or non-DR (NDR).

We used a data augmentation method by using systematically transformed images to augment the class size because of the limited number of data sets. The transformations employed often have to keep the original image’s classification. Each image in the batch could be transformed by operations of random combination in DR and NDR groups in each iteration: (a) horizontal and vertical flips in the case of a random combination of flips and normal images, (b) random small rotations by 90, 180, and 270 degrees, which inherently augment the size of class. In all the network sizes, we used data augmentation during training. Experiments have been conducted utilizing an Intel Core i5-2400 machine running Windows 10 with 4 GB RAM, 160 GB HDD and a Python programming environment.

Many performance indicators were used to assess the system, such as sensitivity (Sens), accuracy (Acc), specificity (Spec), and F1-score. The number of images with DR that were correctly recognized are the number of true positives (TP), divided by the total of TP and false negatives (FP), or images wrongly classified as normal, is represent the sensitivity (recall, or true positive rate). As a result, the sensitivity indicates the percentage of correctly diagnosed DR cases by the system. On the other hand, the normal cases number that is correctly detected or the number of true negatives (TN) and false positives (FP), i.e., images mistakenly classified as DR, divided by the total number of TN and FP are represent the specificity. As a result, specificity is a proportion indicating the percentage of normal cases correctly diagnosed. Precision is the number of correctly predicted positive class values divided by the total number of positive predictions. Finally, the weighted average of recall and precision is the F1-Score. As a result, the F1-score takes into account both FP and FN. F1 is frequently more useful than accuracy, even though it is not as intuitive as accuracy, especially if the class distribution is unequal. When the cost of FP and FN are similar, accuracy works best. If the cost difference between FP and FN is significant, it is best to consider recall and precision [37,38].

**Figure 7:**
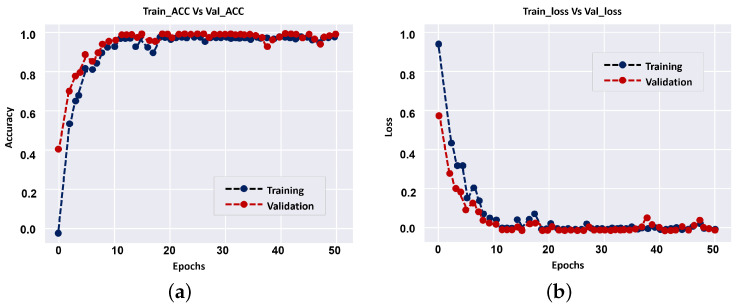
Progression of training and validation set accuracy (**a**) and loss (**b**) during network training.

The results of individual CNN and their fusion are summarized in Table 2. The overall accuracies of DR classification on our dataset for different levels, i.e., 1024 × 1024, 512 × 512, 256 × 256, Fovea (512 × 512), and overall fusion are 72.2%, 83.3%, 88.8%, 88.8% and 94.4%, respectively. The CNN system of fused multi-input outperforms all independent CNN systems in terms of diagnostic accuracy, as shown in Table 2. The results show that employing a smaller number of CNN layers can improve the accuracy of diagnostic, and that is a benefit of the proposed approach over previous CNN techniques. During model training (i.e., 1024 × 1024), Figure 7 shows loss curves and training vs accuracy of the validation. Overall, the results showed that the validation accuracy can achieve 100% with small loss after a few epochs, implying that multi-input CNNs can improve the CAD system’s diagnostic accuracy.

**Figure 8:**
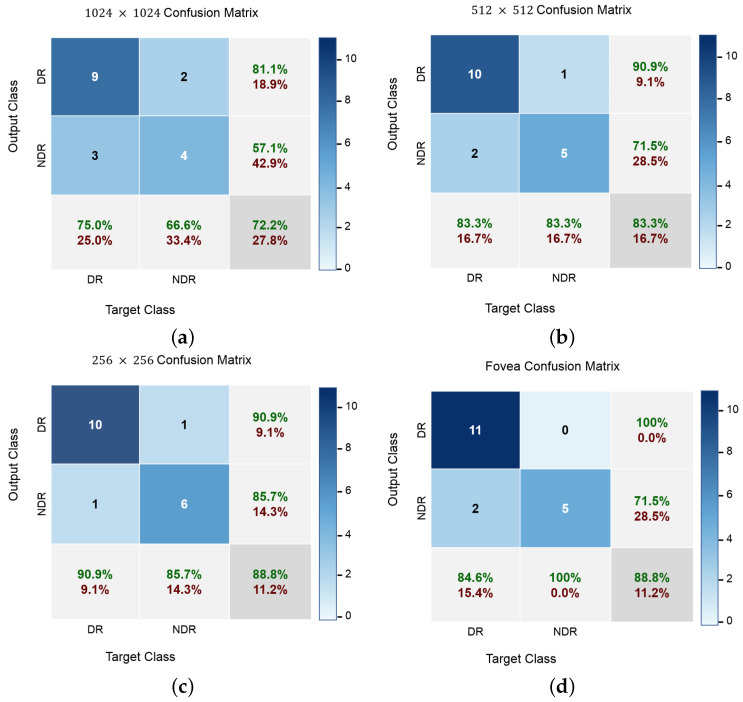
The grading details confusion matrix. The grades DR and NDR respectively correspond to the classes 1 and 2. (**a**) Phase 1: 1025 × 1024; (**b**) Phase 2: 512 × 512; (**c**) Phase 3: 256 × 256; and (**d**) Phase 4: Fovea. Please note that the green and dark-red colored-numbers represent the percentage of correctly and incorrectly classified instances, respectively.

**Figure 9:**
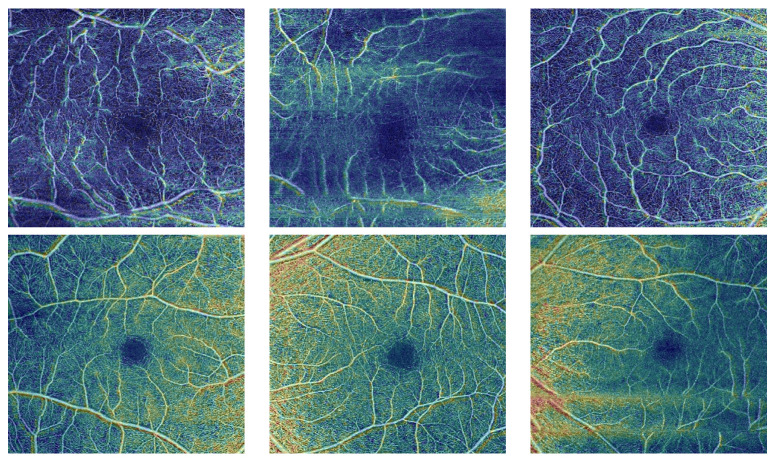
Network attention maps showing the difference between the DR and NDR cases, in the first and second row, respectively.

In addition to accuracy metrics, Figure 8 shows the confusion matrices of the classification results at different input levels. DR cases are easy to distinguish at all input image levels, even though most NDR images are correctly identified. Furthermore, the classification accuracy for NDR and DR is greater when using fovea images, which demonstrates the advantage of a wider visual degree of the retinal range. The original fovea images achieved an accuracy with 88.8% while the images with 1024, 512 and 256 achieved 72.2%, 83.3% and 88.8%, respectively. Since our dataset is not balanced with respect to class size, balanced accuracy is calculated as a weighted kappa. Further, Figure 9 visualises the proposed network attention maps using the visualization model proposed in [39]. The figure clearly shows the difference between the OCTA classes, i.e., DR and DR.

**Figure 10:**
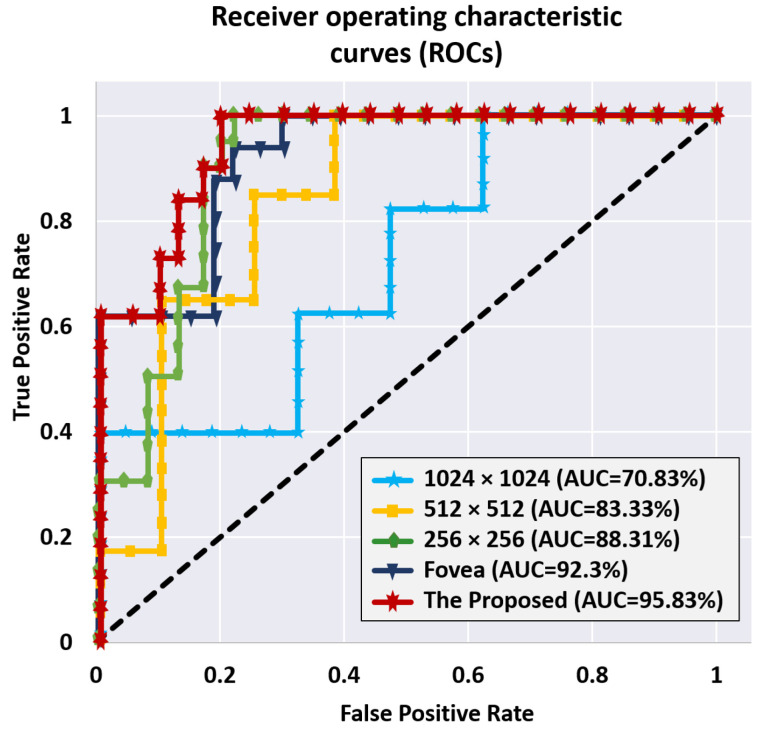
The ROC curves of the proposed CNN classifier for the model cross-validation in all image sizes.

We also used the receiver operating characteristic (ROC) curve to assess the whole system’s accuracy in comparison to the classification threshold setting. This step is necessary to ensure that the used classifier is reliable. The ROC curves for the utilized classifiers at various image levels, as well as their fusion, are shown in Figure 10. The area under a classifier’s respective curve is commonly used to assess its quality (a.k.a. area under the curve or AUC). The classifier is better when AUC is closer to unity. As shown in Figure 10, the AUCs were 70.83%, 83.33%, 88.31%, 92.3%, and 95.83% for the 1024 × 1024, 512 × 512, 256 × 256, Fovea, and total fusion between all classifiers, respectively.

To highlight the advantage of the multi-scale pipeline, comparisons with handcrafted-based ML models, in addition to comparisons with other state-of-the-art CNN approaches for diagnosis of DR have been performed. The results are summarized in in Table 3. As can be seen, our approach achieved an accuracy value of 94.4 compared to 72, 61, 81, 90, 93, 90, 90, 89, and 89 obtained with AlexNet, ResNet 18, random forest (RF), classification tree, K-nearest neighborhood (KNN), support vector machine (SVM Linear), SVM (Polynomial), and SVM (RBF) respectively. In addition, it achieved a sensitivity of 91.7, a specificity of 100, and an AUC of 95.83.

Furthermore, the other state-of-the-art CNN approaches, the methods introduced by Le et al. [28], Alam et al. [30], and Ong et al. [33], tested on their respective dataset, are used for comparison. The proposed CAD system has the best diagnostic performance, according to the comparative results. It is worth noting that, in comparison to the other models, our system has a comparatively low number of layers. It achieved a 94.4(%) overall accuracy, compared with 87.27, 86.75, and 75.2, as shown in Table 4.

## 5. Conclusions and Suggested Future Work

We proposed a novel CAD algorithm to differentiate between DR and NDR. A framework that can detect DR from OCTA based on capturing the appearance and morphological markers of the retinal vascular system. The proposed system’s main contributions are the use of a CNN multi-input that can recognize texture patterns from each input separately. Our CNN model captures significant features to increase its ability to distinguish DR from the normal retina. The proposed approach was tested on in vivo data using 91 patients, which were qualitatively graded by retinal experts. We compared our system’s classification accuracy to that of other deep learning and machine learning methodologies. Our system’s results outperform those produced by competing algorithms. The ultimate goal of our research is to integrate a variety of data types (e.g., OCTA, OCTA, FA, and Funds images), demographic data and standard clinical markers, in order to build a more comprehensive diagnostic system that automates DR grading and diagnosis.

## Figures and Tables

**Table 1:** Summary of our proposed system parameters setting for input size 1024 × 1024.

Layer	Depth	kernel	Stride	Spatial Size	Param.
Input	3	-	-	1024 × 1024 × 3	0
1. Conv	16	3 × 3	1 × 1	1022 × 1022 × 16	432
2. Max-pool	16	2 × 2	2 × 2	511 × 511 × 16	0
3. Conv	32	3 × 3	1 × 1	509 × 509 × 32	4608
4. Max-pool	32	2 × 2	2 × 2	254 × 254 × 32	0
5. Conv	64	3 × 3	1 × 1	252 × 252 × 64	18,432
6. Max-pool	64	2 × 2	2 × 2	126 × 126 × 9	0
7. Conv	128	3 × 3	1 × 1	124 × 124 × 128	73,728
8. Max-pool	128	2 × 2	2 × 2	62 × 62 × 128	0
9. Concat	1	-	-	492,032 × 1	0
10. Full	1	-	-	128 × 1	62,980,096
11. Full	1	-	-	128 × 1	16,384
12. Softmax	1	-	-	128 × 1	0
Batch Size	32				
Learning Rate	0.001				
Optimizer	Adam				
No. of Epochs	50				
Total Parameters	63,095,922				
Trainable Parameters	63,094,930				
Non-Trainable Parameters	992				

**Table 2:** The ACC(%), Sen(%), Spec(%), and F1-score(%) for the proposed DR classifier with multiple size. ACC = accuracy, Sen = sensitivity, and Spec = specificity.

Phases	Acc(%)	Sen(%)	Spec(%)	F1-Score(%)
1024 × 1024	72.2	75.0	66.6	78.3
512 × 512	83.3	83.3	83.3	86.9
256 × 256	88.8	90.9	85.7	90.9
Fovea (512 × 512)	88.8	84.6	100	91.6
Fusion	94.4	91.7	100	95.6

**Table 3:** The proposed DR classification system comparative performance and other related works. Using the ACC (%), Sens (%), Spec (%), and AUC (%).

Tested Systems	ACC (%)	Sens (%)	Spec (%)	AUC (%)
AlexNet	72	80	62	71
ResNet 18	61	70	50	60
RF	81	83	78	87
Classification tree	90	96	78	87
KNN	93	94	91	93
SVM (Linear)	90	96	78	87
SVM (Polynomial)	90	89	91	90
SVM (RBF)	89	96	74	85
Proposed system	94.4	91.7	100	95.83

**Table 4:** Comparisons with the previous works for DR diagnosis.

Study	Data Set Size	Validation (Train:Test)	Technique	ACC
Le et al. [28]	131	(80%:20%)	VGG16	87.27%
Alam et al. [30]	50	(80%:20%)	AV-Net	86.75%
Ong et al. [33]	117	-	DCP VLD-based	75.2%
Proposed model	91	(80%:20%)	Shallow CNN	94.4%

## Data Availability

Data could be made available upon a reasonable request to the corresponding author.
